# Eccentric Compressive Behavior of Round-Ended Rectangular Concrete-Filled Steel Tubes with Different Central Angles

**DOI:** 10.3390/ma15020456

**Published:** 2022-01-07

**Authors:** Zhigang Ren, Qi Li, Gaoyu Wang, Wei Wei, Mohammed A. A. M. Abbas

**Affiliations:** 1 School of Civil Engineering and Architecture, Wuhan University of Technology, Wuhan 430070, China; renzg@whut.edu.cn (Z.R.); wgy159753@Hotmail.com (G.W.); whutweiw@163.com (W.W.); benawadh@whut.edu.cn (M.A.A.M.A.); 2 Hubei Electric Power Survey and Design Institute Co., Wuhan 430040, China; 3 City Investment & Operation Co., Ltd. of China Construction Third Engineering Bureau, Wuhan 430070, China

**Keywords:** concrete-filled steel tube, round-ended rectangular, central angle, eccentric compression, confinement effect

## Abstract

The application of round-ended rectangular concrete-filled steel tubes (RRCFSTs) in high-rise buildings or bridge structures is increasing, improving structural performance and meeting aesthetic requirements. Researching this novel steel–concrete composite helps to fully utilize the properties of the materials. In this study, 15 specimens were tested for analysis of the behaviors of RRCFSTs with different central angles under eccentric compression. Influences of central angles of round ends (*θ*), aspect ratios of rectangular parts (*κ*), steel strength and the eccentric ratio on failure modes, material utilization, confinement effect and eccentric bearing capacity are studied. Besides, the mechanism of confinement effects of steel tubes with different *θ* values was evaluated with the finite element method (FEM). The results show that local buckling usually occurs at the compression zone. When *θ* gradually changes from 0° to 180°, the local buckling position of straight steel plate changes from mid-length to both ends of the columns. Additionally, the interfacial stress between steel tube and concrete at round ends rises, but that at the corner, it decreases continuously, which results in an improved overall confinement effect and increased material utilization. In contrast, a larger *κ* leads to lower material efficiency because of the reduced overall confinement effect. The increases in both *θ* and *κ* enlarge the cross-sectional area and the eccentric ultimate bearing capacity, whereas *θ* has a better influence on the ductility than *κ*. A feasible simplified calculating approach for the eccentric ultimate bearing capacity of RRCFSTs is presented and validated.

## 1. Introduction

Concrete-filled steel tubes (CFSTs) are widely used in bridges and high-rise buildings [1,2,3] due to their excellent strength, ductility, impact resistance and fire resistance. In practice, Round-ended rectangular CFST (RRCFST) columns have been used as pylons in Wuhan Houhu Bridge and side piers in Xiamen Xinglin Bay section [4]. CFSTs can be regarded as a composite material composed of steel and concrete, but unlike traditional fiber composites that enhance concrete bending and impact resistance [5], CFSTs are more concerned with the improvements in compressive performance due to the confined effects of steel tubes. The lateral constraints of external steel tubes under compressive stress substantially improve the strength and ductility of the core concrete [6]. Moreover, the interfacial stress aids to improving the stability of the steel tube and delaying post-local buckling after failure. In addition, CFSTs offer the advantages of simple and lightweight construction, which can result in significant economic benefits; they can also be equipped with internal reinforcement to increase the bearing capacity without increasing the cross-sectional area [7]. Various forms—rectangle [8], round-ended rectangular [9], circular [10], etc.—have been used for the cross-sections of CFSTs, and the confinement effects of steel tubes on core concrete present continuity characteristics with this order [11].

RRCFST concrete columns have the same advantages as rectangular and circular CFSTs. In addition, RRCFSTs have aesthetic benefits [12] and a higher load-bearing capacity as a result of better confinement effects and bending performance due to a greater cross-sectional moment of inertia, making it suitable as a compression–bending load component used in structures [13]. Until now, relevant studies on RRCFST performance are limited, although several engineering applications have been reported [14]. Gu [15] and Xie [16] carried out a study of axial compression tests of RRCFST columns under different parameters, and the analysis shows the confinement effects of round-ended steel tubes on the core concrete between the circular and rectangular steel tubes. Hassanein [17] divided the round-ended rectangle cross-section into a rectangular part and a semicircle at both ends. The concrete in the two parts was simulated with different stress–strain relationships by the finite element method and compared with the axial compression test results; the findings showed that the finite element method can simulate the compressive properties of RRCFST columns. Zhang [18] used the fiber model method to investigate the constitutive relationship of concrete in RRCFSTs, which was determined to be appropriate to the case of thin-walled steel tubes by comparison with tests and to explain the observed stiffness degradation behavior. Shen [9] and Wang [19] proposed an equivalent constitutive model for core concrete in an RRCFST, which equates the core concrete to a circular CFST and a rectangular CFST at smaller and larger aspect ratios, respectively. Their research showed that circular and rectangular tubes can be treated as RRCFSTs with extreme parameters. In addition to the traditional test methods, digital image correlation (DIC) is a relatively advanced digital image recognition technique that can be used to evaluate the deformation of steel–concrete structures, but the current research focuses on the fine-scale behavior of the studied objects. For example, Grzegorz [20] used the DIC technique to observe the fracture behavior of fly ash concrete during shear.

**Figure 1:**
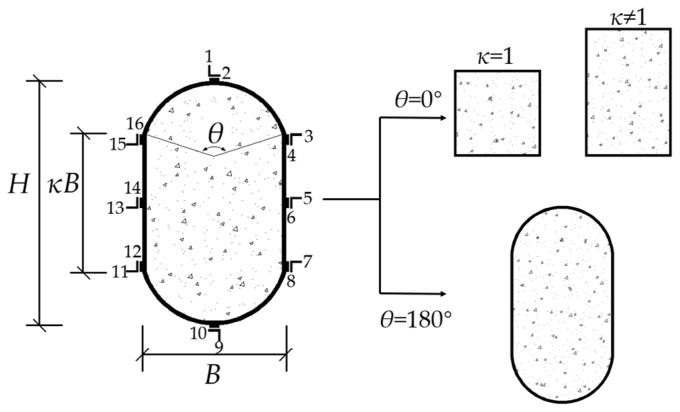
Cross-section of an RRCFST.

Current literature research on RRCFSTs is primarily focused on numerical analysis; however, more original experimental research is required to enable actual engineering applications. In addition, two round ends of RRCFSTs are usually adopted as two semi-circles, with a central angle of 180°. As a novel form of steel–concrete combination structure, however, only Ren and Wang [13] have studied the axial compression performance of RRCFSTs with two arches at the circular end, i.e., with a circular center angle, *θ*, between 0° and 180°, as illustrated in Figure 1. There is still a lack experimental studies on RRCFSTs with different central angles and columns which withstand the eccentric loads in engineering practice [21]. The study of the mechanical properties of RRCFSTs under eccentric compression, and the effects of multiple parameters on the strength utilization efficiency of steel tubes and concrete can further promote the application of CFSTs in high-rise buildings and large bridges. Therefore, the behavior of RRCFST columns with different central angles under eccentric compression was researched in this study.

This study aimed to research the eccentric compressive behavior of RRCFSTs by experimental and finite element methods and analyze the unique confinement effects of steel tubes on the concrete. Building on previous studies on the axial performance of RRCFSTs with different central angles, 15 stub columns (*L*/*H* ≈ 3) were designed and tested under eccentric load with different central angles, aspect ratios, steel strength and eccentricity ratios. The changes in the failure mode and mechanical properties of RRCFST columns with different parameters under eccentric pressure were investigated. Quantitative relationships between the above parameters and material utilization were obtained. The effects of variations in geometric parameters on the confinement effects of steel tubes were investigated by contact stress from the finite element method, and the relationships between the confined effect and the failure mode, mechanical properties and material utilization of RRCFST columns are further elaborated. Furthermore, a simplified eccentric bearing capacity calculation method of RRCFSTs is presented and validated.

## 2. Research Methods

### 2.1. Experimental Program

#### 2.1.1. Specimens Preparation

15 eccentric compressive specimens were tested in this research. PYRE1-60-e1 and PYRE1-120-e1 were set for ensuring the accuracy of PY1-60-e1 and PY1-120-e1. Figure 1 shows the cross-section of the specimen. Sectional shape functions *S*(*κ*, *θ*) consist of the aspect ratio (*κ*) in the rectangular part and central angle (*θ*). Additionally, 16 strain gauges were placed at the middle section of the specimen to monitor longitudinal and transverse strains, but most of them were broken during the experiments; only axial strains of PY0.5-180-e2 and PY1-180-e1-Q235 were saved. The measurement results of the dimensions of RRCFSTs, including height (*H*), width (*B*) of the cross-section and length (*L*) of the column, are shown in Table 1, and two eccentricity ratios (*e*/2*i* = 0.15, 0.3, where *e* is the eccentricity distance and *i* is the radius of gyration for the cross-section) were designed for testing the bearing capacity of the RRCFST column under different combinations of moment and axial load. All specimens had a steel tube thickness of 3.4 mm.

Based on Chinese code specifications for mix proportion designs of ordinary concrete—JGJ 55-2011 [22]—the mix proportions of concrete with design strength C30 were: P.O 42.5 cement (391 kg/m^3^); sand (677 kg/m^3^); aggregate (1152 kg/m^3^); water (180 kg/m^3^) in a 1:1.73:2.95:0.46 ratio. The mean cubic compressive strength, *f*_cu_, was 30 MPa. Three cylinders with a diameter of 150 mm and a height of 300 mm were used to conduct the axial compressive experiment and provided the mean cylindrical strength $f_{c}^{\prime }=$ 25.7 MPa.

Q235 steel was used to produce the steel tubes for columns identified with Q235, and Q345 was used for the other samples. In accordance with Chinese Standard GB2975, the main properties of the steel are shown in Table 2.

#### 2.1.2. Test Method

**Figure 2:**
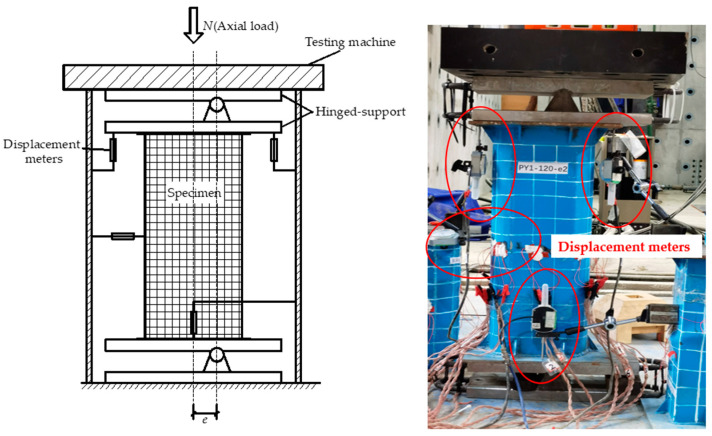
Test setup.

Figure 2 shows the part of the specimens during preparations and the WAW-J10000 electro-hydraulic servo multifunction structure testing machine. The distances between the top-hinged support and the centerline of the testing machine, and the bottom-hinged support and projection on the ground of the centerline of the testing machine were measured to ensure the correct location of eccentric loads. The displacement loading with an initial speed of 0.5 mm/min was adopted and increased to 1 mm/min after the testing machine had been stabilized. The speed was slowed down to 0.3 mm/min when it neared the bearing capacity, to prevent lateral overturn of the specimen due to sudden unloading. The load was stopped when the weld cracked or axial deformation reached 3%. Three displacement meters were used to measure the displacement of the top and bottom hinged supports; the deflection of the specimen at the mid-length of the specimen was measured by one displacement meter.

### 2.2. Finite Element Method (FEM)

The finite element method (FEM) was used for mutual authentication with experiments and a supplement for RRCFSTs with multiple parameter research, which was established in ABAQUS finite element software. Research on the behavior of concrete under complex constraints was well established by Han et al. [6]. Depending on the confined effect of steel tubes and concrete, concrete at the round-ended part and rectangular part was simulated with different constitutive models, such as provided by Han [6], and is described as:

(1) $$y=\left\{ \begin{array}{l} 2{x-x}^{2}\;\;\;\;\;\;x\leq 1\\ \frac{x}{\beta_{0}{\left(x-1\right)}^{\eta }+x}\;\;\;x>1 \end{array}\right.$$

of which:

(2) $$y=\frac{\sigma }{\sigma_{0}};\;x=\frac{\varepsilon }{\varepsilon_{0}}$$

(3) $$\sigma_{0}{=f}_{c}^{\prime }$$

(4) $$\varepsilon_{0}=\varepsilon_{c}+800\xi^{0.2}\cdot 10^{-6}$$

(5) $$\varepsilon_{c}=\left(1300+12.5f_{c}^{\prime }\right)\cdot 10^{-6}$$

(6) $$\xi =\frac{f_{y}A_{s}}{f_{c}A_{c}}$$

(7) $$\beta_{0}=\left\{ \begin{array}{l} {\left(2.36\times 10^{-5}\right)}^{\left[0.25+{\left(\xi -0.5\right)}^{7}\right]}\cdot {\left(f_{c}^{\prime }\right)}^{0.5}\cdot 0.5(\mathrm{Round}-\mathrm{ended})\\ \frac{{\left(f_{c}^{\prime }\right)}^{0.1}}{1.2\sqrt{1+\xi }}\;\;\;\;\;\;\;\;\;\;\;\;\;\;\;\;\;\;\;\;\;\;\;\;\;\;\;\;\;\;\;\;\;\;\;\;\;\;\;\;\;\;\;\;\;(\mathrm{Rectangular}) \end{array}\right.$$

(8) $$\eta =\left\{ \begin{array}{l} 2\;\;\;\;\;\;\;\;\;\;\;\;\;\;\;\;\;\;\;\;\;\;\;\;(\mathrm{Round}-\mathrm{ended})\\ 1.6+1.5/x\;\;\;\;\;\;\;(\mathrm{Rectangular}) \end{array}\right.$$

where *σ* and *ε* are the axial stress and strain of the concrete, respectively, and *ξ* is the confined coefficient of the steel tube.

In ABAQUS, the plastic damage of concrete was simulated by the CDP (concrete damage plastic) model with a damage plastic factor, *d*. China code GB50010-2010 [23] indicates that a damage plastic parameter *d*_c_ is not suitable for a CDP model [24]; however, an equation between *d* and *d*_c_ was proposed by the Sidoroff energy equivalent theory [25,26], which is expressed as:

(9) $$d=1-\sqrt{1-d_{c}}$$

of which:

(10) $$d_{c}=1-\frac{\sigma }{E_{c}\varepsilon }$$

**Figure 3:**
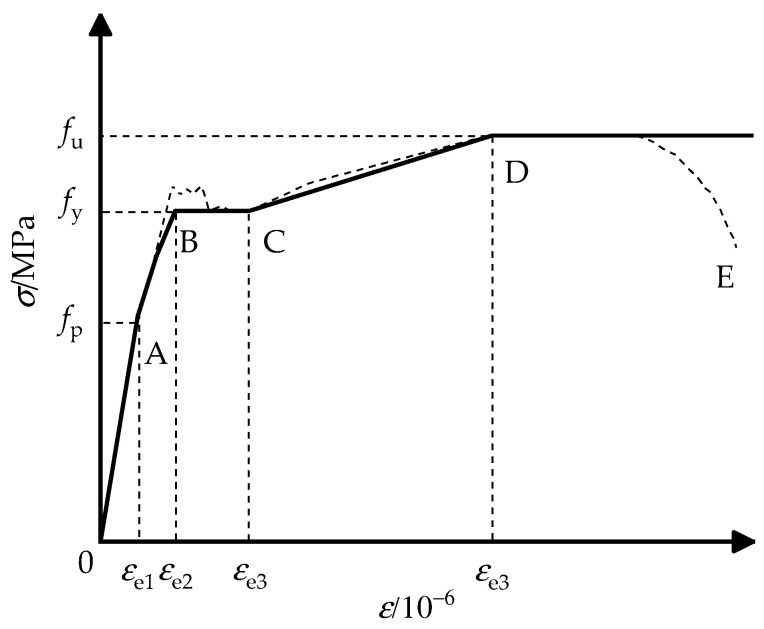
Stress–strain relationship of the steel tube.

The tensile softening model from reference [15] was adopted for simulating the property of concrete under tension. As demonstrated in Figure 3, the stress–strain relationship [6] of steel proposed by Han includes 5 stages: OA for the elastic stage, AB for the elastic–plastic stage, BC for the plastic stage, CD for the strain hardening stage, and final plastic deformation after point D.

**Figure 4:**
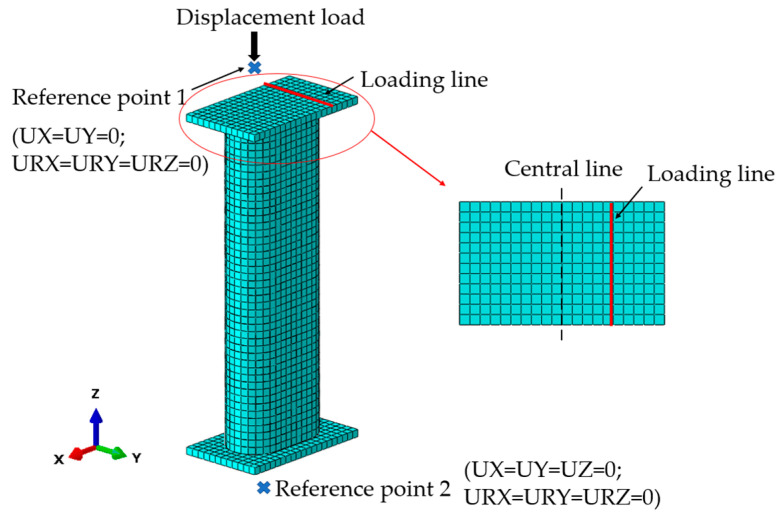
Finite element model.

The FEM was established in ABAQUS. A 3D 8-node solid element, C3D8R, was selected for concrete and the rigid plate; a 3D 4-node shell element, S4R, was selected for the steel tube. “Surface-to-surface” was selected for interactions between concrete and the steel tube, a penalty function with a 0.6 friction coeffective was used for the tangential behavior, and “Hard” contact for the normal behavior. “Shell-to-solid-coupling” and “tie” were applied for the rigid plate with the steel tube and concrete, respectively. A mesh size of 15 mm was adopted for the hole model to ensure accuracy and calculate speed. After the mesh procedure, 19,036 to 23,248 nodes and 15,608 to 19,464 elements were produced. Figure 4 displays the FE model; the eccentric loading was simulated with boundary conditions, the loading line was coupled with a reference point which was constrained in the x and y directions so that it could move in the z-direction, and the plate at the bottom of the column was constrained in x, y and z directions.

## 3. Results and Discussion

**Figure 5:**
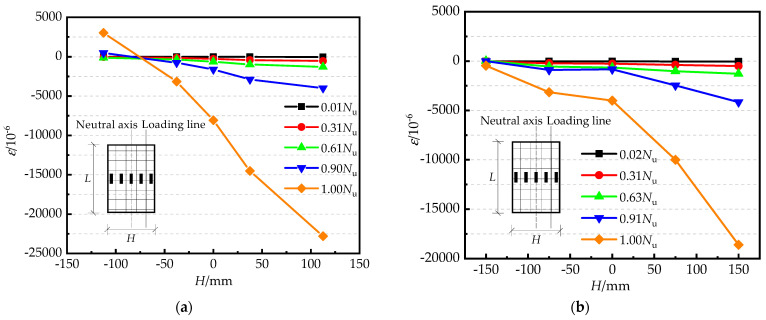
The strain curves of the normal section of the RRCFST columns: (**a**) PY0.5−180−e2; (**b**) PY1−180−e1−Q235.

Figure 5a,b presents the axial strain along with the height of the cross-sections at the mid-length of specimens PY0.5-180-e2 and PY1-180-e1-Q235. The changes in the steel tube strain along the normal section of specimens confirm the plane-section assumption [27]. A large increase in strain occurred at loads at 1.00 *N*_u_, which possibly corresponded with the development of local buckling at the mid-length.

**Figure 6:**
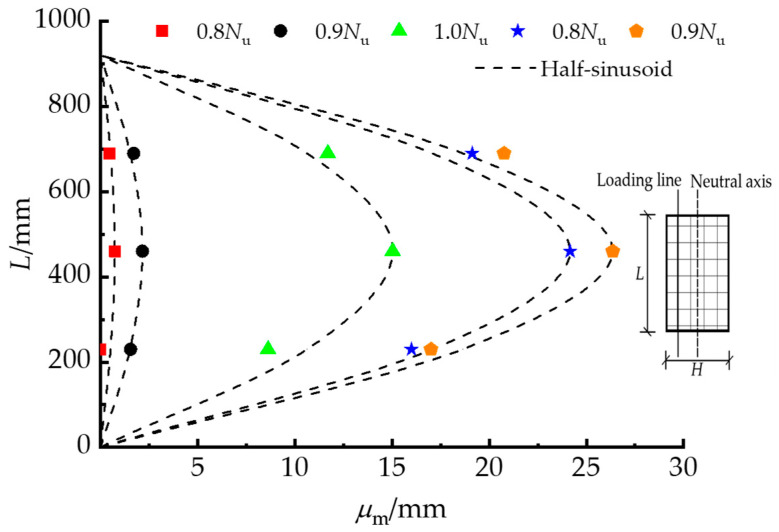
Lateral deflection curve of the typical specimen.

Figure 6 displays a typical lateral deflection curve for PY1-180-e1-Q235 at various load levels; *μ*_m_ is the lateral displacement and *N*_u_ is the peak axial load. The curves were in good agreement with the half-sinusoid curves. It is feasible to predict the peak load and lateral deflection of the RRCFST columns under eccentric compression.

### 3.1. Failure Model

**Figure 7:**
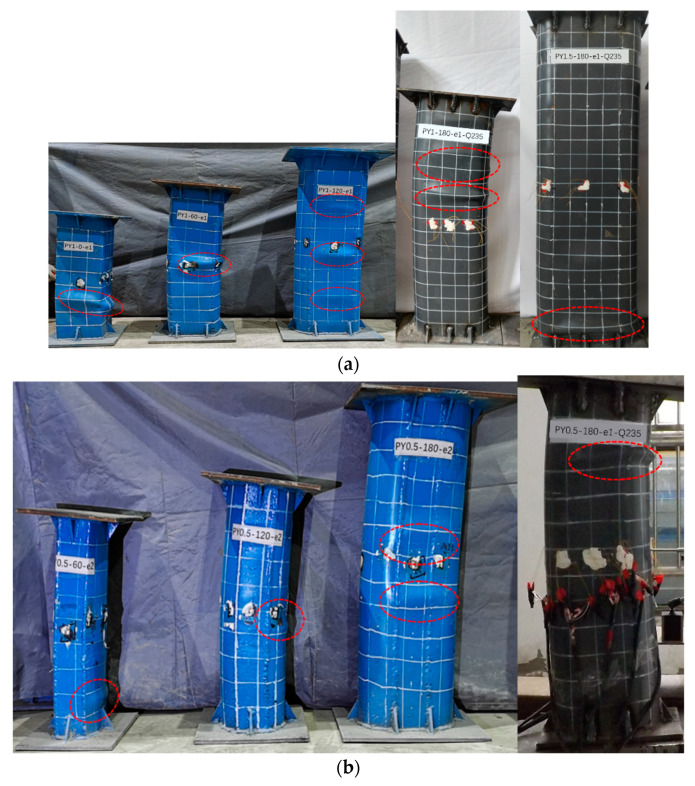

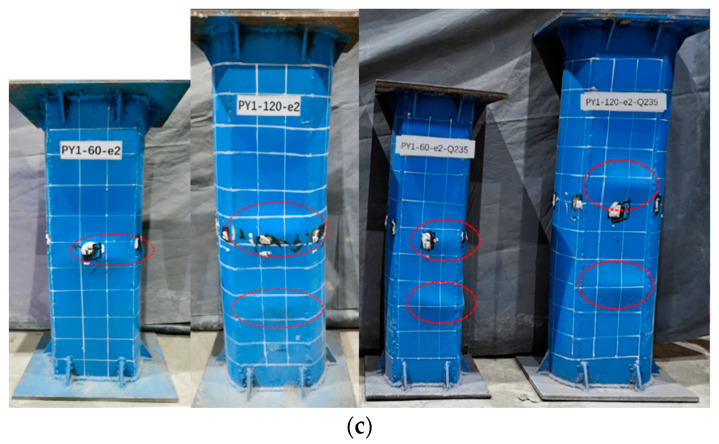
Failure modes: (**a**) *κ* = 1 and 1.5, with different *θ* and *f*_y_; (**b**) *κ* = 0.5, with different *θ* and eccentric ratio; (**c**) Different *f*_y_.

Figure 7 gives the failure modes of columns at the front and corresponding compressive side after eccentric compression experiments. Most eccentric compression specimens fail due to the steel tube buckling and crushing of the concrete. However, the shear failure mode is observed in PY1-0-e1 with weak confinement by the outer thin-walled steel tube [19,28]. For specimens in Figure 7a, with central angles from 0 to 180°, local buckling developed from the mid-length to both ends and from the round-ended part to the rectangular part. This was probably due to the increased local confinement effect at the round ends in the presence of a large *θ*. The confined effect of different central angles is discussed in Section 3.4. In addition, when the central angle was increased from 120° to 180°, the lower steel strength had only a minor impact on the lateral deformation and failure mode of the RRCFST. However, buckling still occurred at the round-ended parts of PY1-180-e1-Q235 and PY1.5-180-e1-Q235, demonstrating that a larger central angle generates a more contained effect, but it is insufficient to compensate for the reduced material strength. According to the failure mode results in this research, these regular buckling types of RRCFST stub column failure which can be observed are attributed to the influence by the regular *θ* changes.

Comparing the failure modes of PY0.5-180-e1-Q235, PY1-180-e1-Q235 and PY1.5-180-e1-Q235 in Figure 7b, the lateral stiffness of columns with the same *θ* value are enhanced with the increased cross-sectional area, although local buckling still occurred at the end because of stress concentration. However, a narrower range of local buckling and lower buckling amplitude occurred in columns in Figure 7b compared with the columns in Figure 7a. It is hypothesized that a lower aspect ratio contributes to delaying the local buckling and improves the confinement of steel tube on concrete.

As shown in Figure 7c, the enhanced strength of the steel tube resulted in improvements for local buckling, and benefited the lateral deformation. Similarly, this relationship between central angles and steel strength and the influence on failure modes is displayed in Figure 7a. Therefore, the optimum parameter combination (*θ,* *κ*, *f*_y_) is required for proper material use.

### 3.2. Axial Load and Mid-Length Lateral Deformation Relationship

**Figure 8:**
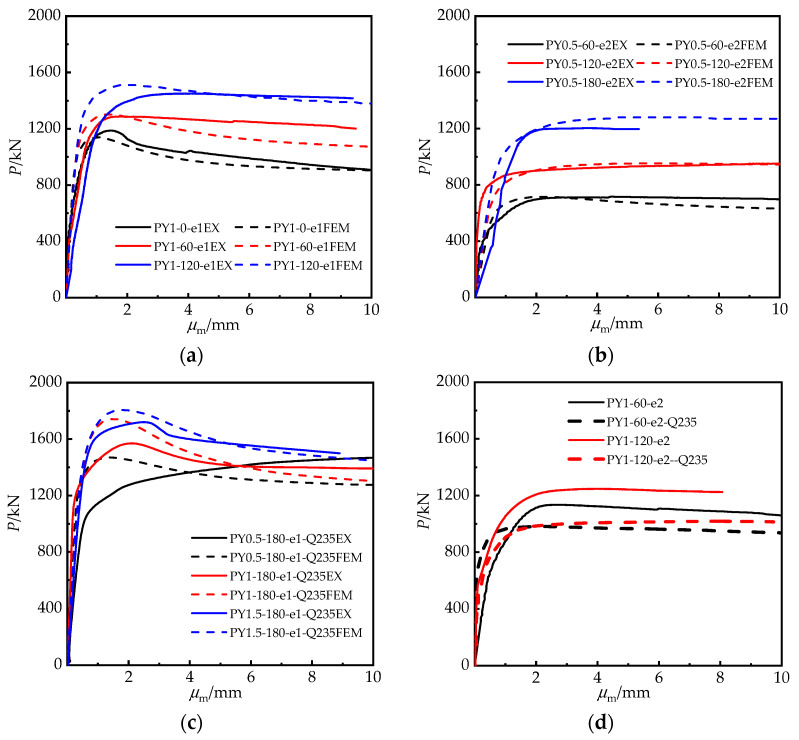
Axial load vs. mid-length lateral displacement curves: (**a**) *κ* = 1, with different *θ*; (**b**) *κ* = 0.5, with different *θ*; (**c**) *θ* = 180°, with different *θ*; (**d**) Different *f*_y_.

The axial load (*P*/kN) and mid-length lateral displacement (*μ*_m_) curves are exhibited in Figure 8. The initials EX and FEM stand for experimental and finite element model findings, respectively. FEM ignores the specimen’s original defects; thus, the FEM curves in the elastic–plastic range are somewhat higher than the experimental curves in Figure 8a–c.

Due to the increasing cross-sectional area of the specimen, when the central angle rose from 0° to 120°, the specimen’s eccentric bearing capacity and residual bearing capacity were undoubtedly enhanced. Moreover, the ductility improved as the descending part of the curve flattened out. With the increase in *θ*, the area under the curve wrapping likewise increased, which indicated that the ability of the RRCFST columns to absorb energy also improved. The data in Figure 8b demonstrate this as well. The cross-sections of the specimens in Figure 8b tended to be more circular, and the confinement effect provided by the steel tube becomes more obvious as the central angle rises, resulting in a smoother descending section of the curves and improved ductility of the RRCFST column.

The results in Figure 8c show that the confinement effect of the steel tube was enhanced with the increased aspect ratio of the cross-section *κ* at the same central angle. Indeed, the bearing capacity of specimen PY0.5-180-e1-Q235 with a smaller eccentric ratio of 0.15 increased after yielding due to the high confinement effect. The curves after the peak fell more steeply as *κ* increased, illustrating that the confinement effect of steel tube on concrete influenced the specimen’s ductility as well as its bearing capacity.

The test results of specimens with varying steel strengths are shown in Figure 8d. The statistics suggest that increasing the steel tube strength is a simple way to increase the eccentricity bearing capability and ductility of RRCFST columns. Moreover, the falling area of the *P*–*μ*_m_ curves for specimens with an eccentric ratio of 0.3 and central angles of 60° or 120° exhibited the same characteristics as in Figure 8a, implying that the eccentric ratio did not influence the RRCFST column deformation law.

In general, the displacement ductility coefficient is used to assess the deformation ability of CFSTs after yield [6]. However, the axial load–lateral displacement curves of PY1-0-e1, PY1-180-e1-Q235 and PY1.5-180-e1-Q235 exhibited a downward trend after the columns yielded, whereas others show a flat or even increasing trend. Therefore, typical ductility quantification methods are ineffective for RRCFSTs under static load, and additional tests are necessary to evaluate its performance and response during seismic activity.

The peak loads (*N*_u_/kN) for the FEM (*N*_uf_/kN) and experiment (*N*_ue_/kN) specimens are compared in Table 3. The average ratio of *N*_uf_/*N*_ue_ is 1.014, with a dispersion coefficient of 0.042. A good agreement is presented between the finite element model and experiment results of eccentric bearing capacity.

### 3.3. Parameter Analysis on Material Utilization

Finite element models were established to support the experimental results. Furthermore, material utilization was analyzed to further study the key parameters in the RRCFST stub column design. The strength index, *SI*, was utilized to assess the effectiveness of employing the complete plastic compressive resistance of concrete and steel tube materials, and expressed in Equation (11) [29]:

(11) $$\mathrm{SI}=N_{u}/N_{p}$$

where *N*_p_ is the plastic compressive resistance of the column cross-section, and can be defined as follows [30]:

(12) $$N_{p}=A_{c}f_{c}^{\prime }+A_{s}f_{y}$$

**Figure 9:**
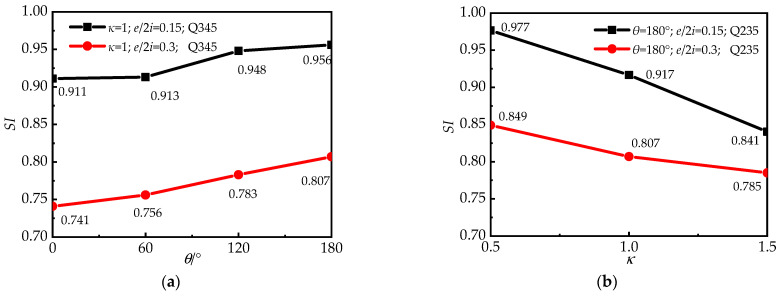

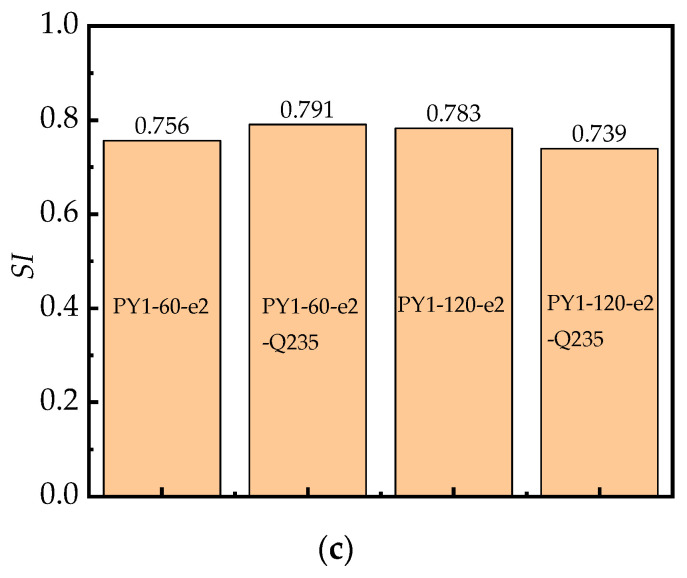
*SI* for columns with different paraments: (**a**) central angle, *θ*; (**b**) aspect ratio of cross-section, *κ*; (**c**) strength of steel tube, *f*_y_.

Figure 9 depicts the *SI* of each specimen based on several parameters. As can be seen in Figure 9a, the *SI* increased as the degree of the central angle increased. *SI*, i.e., material utilization, rose by 0.2 to 3% for every 60° increase in central angle, which was particularly noticeable when the eccentricity was large. This also shows that the confinement effect in the round-ended part and the material efficiencies of steel and concrete in RRCFST columns improved with the increased central angle when they are subjected to eccentric load. On the other hand, Figure 9b shows how differing cross-sectional aspect ratios affect the *SI*. The cross-section tends to be rectangular when *κ* is raised, resulting in fewer constraints for the steel tube and concrete and a loss in material efficiency; the SI reduces by 2.7% to 8.3% for a 0.5 increase in aspect ratio. Figure 9a,b demonstrates the concept that the cross-section tends to be circular when the aspect ratio falls with the central angle increase and leads to enhanced confinement effects of concrete from the steel pipe, and high material utilization. Furthermore, with the increased eccentric ratio, the bearing capacity of columns is diminished due to the combined effect of axial load and enlarged moment. Moreover, the trend in *SI* is less affected by an eccentric ratio as an external cause. With a different eccentric ratio, the change in *SI* remains higher as the central angle increases and the aspect ratio decreases.

*SI* results for columns with various steel strengths are presented in Figure 9c. The results reveal an unknown regular pattern for *SI* as the steel strength changes. This is probably due to the strong association between the steel tube and concrete. Material utilization does not change substantially with minimal variation in the strength of concrete and steel tube. As a result, for the design of RRCFST columns, the strong relationship between the steel tube and concrete should be further investigated.

### 3.4. Confinement Effect

**Figure 10:**
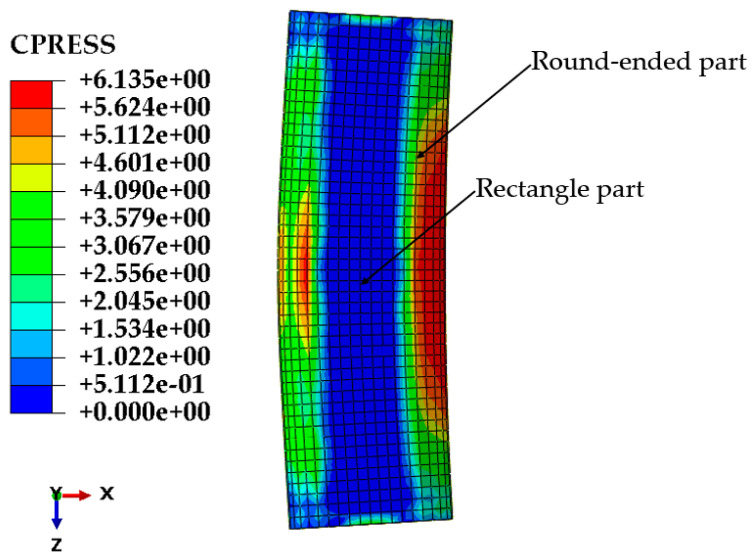
Interfacial stress of PY1-180-e1-Q235.

The eccentric bearing capacity of RRCFST columns greatly depends on the confinement effect of the steel tube on concrete, and the confinement effect can be quantitatively illustrated by the interfacial stress between the steel tube and concrete [19]. However, the interfacial stress was hard to measure in this experiment. In this study, “contact stress” from the FEM is used to represent the interfacial stress between steel tube and concrete, and the confinement effect is analyzed. Based on the FEM results, the interfacial stress contour distributions of PY1-180-e1-Q235 have been produced and are destroyed in Figure 10. The biggest stress occurs at the compressive area and the corner where the straight and arc edges join. The large region in the rectangular part contains similar and comparable interfacial stress.

**Figure 11:**
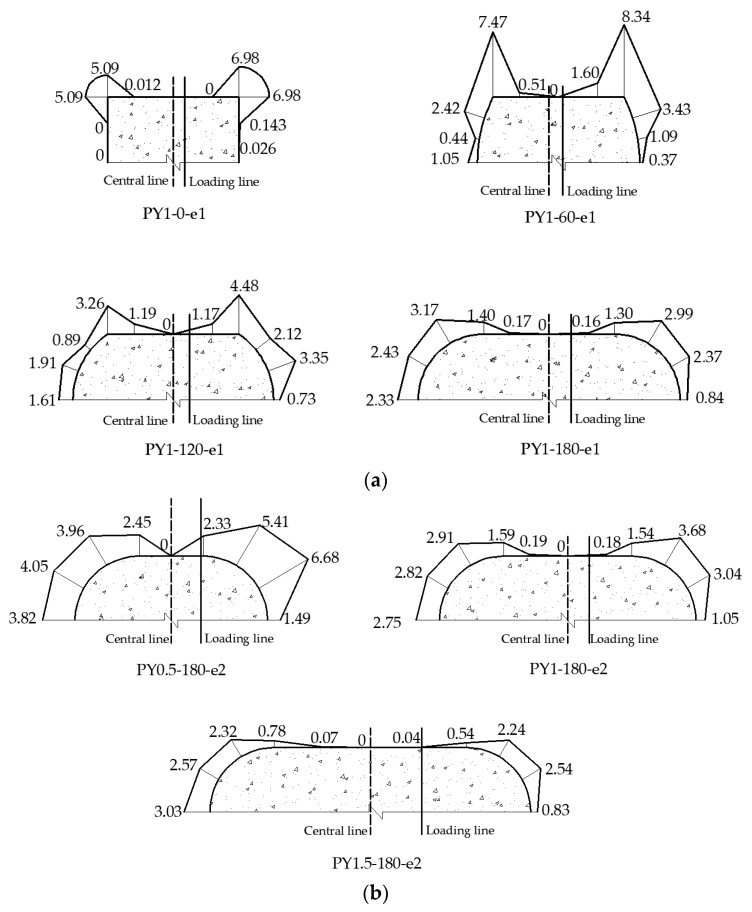
Contact stress between steel tube and concrete at mid-length for RRCFST columns with different parameters: (**a**) central angle; (**b**) aspect ratio.

Quantitative contact stress from the FEM of concrete from the steel tube at peak load is plotted as shown in Figure 11. Half of the mid-length segment was employed for the analysis due to the symmetry of the columns. When the central angle was 0°, the cross-section took the shape of a square, and the steel tube constraint occurred mostly at the corner, as illustrated in Figure 11a. When the RRCFST column was under tension, the existence of contact stress between concrete and the steel tube in the tensioned area implies that there was a mutual constraint in this interface. The corner constraint was more consistently distributed in the RRCFST columns with the same aspect ratio (*κ* = 1), and it shifted to the round-ended section as the central angle increased. When the column was exposed to eccentric loading, the contact stresses at the neutral axis were usually minor or even unobservable, suggesting that there is less mutual restriction between the lateral steel tube and concrete. This is also illustrated by the fact that steel tube buckling always occurred in the rectangular part, shown in Figure 7.

Figure 11b shows that when the RRCFST columns had the same central angle (*θ* = 180°), the contact stress at the round-ended part decreased as the section aspect ratio increased, but the contact stress at the rectangular part remains low, indicating that the confinement effect of steel tube on concrete decreased as the aspect ratio increased. The steel tube confinement effect was optimized when the aspect ratio was minimal and the shape of the cross-section was approximately circular.

### 3.5. Calculation and Verified of Eccentric Compression Bearing Capacity

To investigate the eccentric compression bearing capacity of RRCFST columns and the influence of the type of cross-section, a simplified calculation method based on China code CECS28-2012 was studied in this paper, which can be expressed as:

(13) $$N_{u1}=\varphi_{1}\varphi_{e}N_{\mathrm{ua}}$$

where *φ*_1_ is the reduction factor of the slenderness ratio, equal 1.0 for stub columns, *φ*_e_ is reduction factor of eccentric ratio which is fitting by the experimental results and can be expressed as Equation (14):

(14) $$\varphi_{e}=\left\{ \begin{array}{l} \frac{1}{1+0.98(e/2i)}\;\;\;\;\;\;\;\;\;\;\;\;\;\;\;e/2i<0.8\\ \frac{0.45}{e/2i}\;\;\;\;\;\;\;\;\;\;\;\;\;\;\;\;\;\;\;e/2i\geq 0.8 \end{array}\right.$$

*N*_ua_ is the axial bearing capacity of RRCFST columns with different central angle, which had been deduced in reference [31], and determined by:

(15) $$N_{\mathrm{ua}}=f_{c}A_{c}+f_{y}A_{s}+5.3t^{2}f_{y}\sqrt{\kappa +0.5x}$$

of which: $x=\frac{\theta -\sin\theta }{1-\cos\theta },\theta >0{}^{\circ}$.

**Figure 12:**
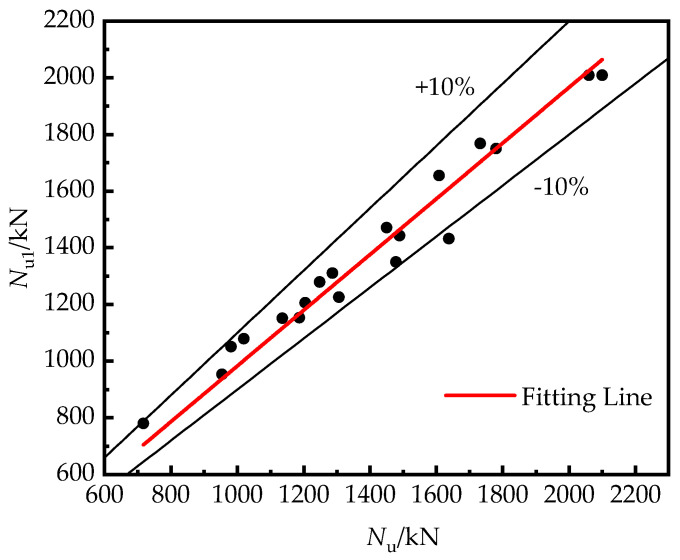
Fitting relationship between experimental (*N*_u_) and calculation (*N*_u1_) results.

Due to the lack of eccentric bearing capacity for RRCFST columns with different central angles, the experimental results in this study were used for examining the feasibility of Equation (9), and are described in Figure 12. A good fitting level is exhibited. This provides a simple calculation method for presenting the bearing capacity of RRCFST stub columns with different central angles under eccentric compression load.

## 4. Conclusions

This paper proposes a novel concept of RRCFST stub columns with different central angles. Assessments of the eccentric compressive performance and confinement effects of RRCFST stub columns with key parameters were carried out through experiments and FEM. Based on the current study, the following conclusions can be drawn:

(1) The typical failure mode of RRCFST stub columns is buckling of the steel tube in the compression zone; this local buckling is accompanied by concrete shear damage when the *θ* is minor. Increased *θ*, reduced *κ* and enhanced *f*_y_ contribute to improving the local buckling resistance of RRCFSTs, while optimizing the design parameter combinations between them needs further study;
(2) Specimens in FEM and experiment reveal similar deformation and bearing capacity, which represents another possible method to study the eccentric performance of RRCFSTs in addition to experiments: numerical simulations;
(3) Typical ductility quantification methods are ineffective for RRCFSTs under static load due to the favorable post-yield deformation ability, and additional tests are necessary to evaluate their performance and response during dynamic activity;
(4) Material efficiency can be improved with an increased *θ* and a reduced *κ*. *SI*, i.e., material utilization, rises by 0.2% to 3% for every 60° increase in *θ*, which is particularly noticeable with a large eccentricity. And it reduces by 2.7% to 8.3% for a 0.5 increase in aspect ratio, but there is an unknown tendency as the steel strength changes. *SI* is always less than 1.0 when axial force and bending moment work together;
(5) Study of the confined effect verified the assumptions discussed in the failure modes. A smaller *κ* and larger *θ* contribute to enhancing the confinement effect at the round-ended part, resulting in less local buckling occurring at columns. However, local buckling still occurred in the rectangular part due to the weaker interaction relationship between the steel tube and concrete;
(6) Current codes cannot be used to calculate the eccentric ultimate bearing capacity of RRCFST stub columns with different center angles. A simplified calculating approach has been demonstrated and validated in this study.

## Figures and Tables

**Table 1:** Main parameters of specimens.

Identifier	*H × B × L*/mm^3^	(*κ*, *θ*)	*i*/mm	*e*/2*i*	*e*/mm
PY1-0-e1	153 × 148 × 450	(1, 0)	43.35	0.15	13.01
PY1-60-e1	189 × 153 × 550	(1, 60)	51.48	0.15	15.44
PYRE1-60-e1	187 × 151 × 550	(1, 60)	51.48	0.15	15.44
PY1-120-e1	232 × 152 × 650	(1, 120)	62.19	0.15	18.66
PYRE1-120-e1	235 × 150 × 650	(1, 120)	62.19	0.15	18.66
PY0.5-180-e1-Q235	225 × 150 × 675	(1, 180)	57.96	0.15	17.39
PY1-180-e1-Q235	300 × 150 × 920	(1, 180)	79.02	0.15	25
PY1.5-180-e1-Q235	375 × 150 × 1160	(1.5, 180)	100.31	0.15	30.09
PY1-60-e2	188 × 152 × 550	(1, 60)	51.48	0.3	30.89
PY1-60-e2-Q235	188 × 152 × 550	(1, 60)	51.48	0.3	30.89
PY1-120-e2	237 × 150 × 650	(1, 120)	62.19	0.3	37.31
PY1-120-e2-Q235	235 × 150 × 650	(1, 120)	62.19	0.3	37.31
PY0.5-60-e2	110 × 153 × 445	(0.5, 60)	30.1	0.3	18.06
PY0.5-120-e2	156 × 155 × 500	(0.5, 120)	41	0.3	24.60
PY0.5-180-e2	224 × 152 × 650	(0.5, 180)	58	0.3	34.80

**Table 2:** Steel properties.

Identifier	*f*_y_/MPa	*f*_u_/MPa	*E*_s_/GPa	*γ*
Q235	279	418.5	225	0.27
Q345	361	541.5	240	0.27

**Table 3:** Comparison of FEM and experiment peak loads.

Identifier	*N*_uf_	*N*_ue_	Ratio (*N*_uf_/*N*_ue_)
PY1-0-e1	1140	1187	0.960
PY1-60-e1	1301	1287	1.011
PYRE1-60-e1	1301	1306	0.996
PY1-120-e1	1510	1450	1.041
PYRE1-120-e1	1510	1489	1.014
PY0.5-180-e1-Q235	1406	1478	0.951
PY1-180-e1-Q235	1758	1638	1.073
PY1.5-180-e1-Q235	1825	1732	1.054
PY1-60-e2	1086	1136	0.956
PY1-60-e2-Q235	957	981	0.976
PY1-120-e2	1275	1248	1.022
PY1-120-e2-Q235	1116	1020	1.094
PY0.5-60-e2	715	717	0.997
PY0.5-120-e2	952	954	0.998
PY0.5-180-e2	1278	1205	1.061

## Data Availability

The data presented in this study are available upon request from the corresponding author.
